# Association of weight gain and fifteen adipokines with declining beta-cell function in Mexican Americans

**DOI:** 10.1371/journal.pone.0201568

**Published:** 2018-08-13

**Authors:** Anny H. Xiang, Mary Helen Black, Yu-Hsiang Shu, Jun Wu, Adrienne MacKay, Corinna Koebnick, Richard M. Watanabe, Thomas A. Buchanan

**Affiliations:** 1 Department of Research & Evaluation, Kaiser Permanente Southern California, Pasadena, CA, United States of America; 2 Department of Preventive Medicine, Keck School of Medicine of USC, Los Angeles, CA, United States of America; 3 Diabetes and Obesity Research Institute, University of Southern California, Los Angeles, CA, United States of America; 4 Department of Medicine, Keck School of Medicine of USC, Los Angeles, CA, United States of America; SERGAS and IDIS, SPAIN

## Abstract

Obesity and adipokines are associated with development of type 2 diabetes. However, limited longitudinal studies have examined their roles on declining β-cell function over time. This report assessed three adiposity measures (BMI, percent body fat, trunk fat), insulin resistance, and fifteen adipokines in relationship to longitudinal change in β-cell function measured by disposition index (DI) from frequently-sampled-intravenous-glucose-tolerance testing. The results showed that three factors were significantly and independently associated with rate of change in DI over time: rate of change in BMI (negative), rate of change in IL-6 (negative), and baseline adiponectin (positive). The association was the strongest for changing BMI and was largely explained by changing insulin resistance; the association with changing IL-6 was also largely explained by changing insulin resistance. Baseline adiponectin remained positively associated after adjustment for changing insulin resistance, suggesting an independent effect of adiponectin to preserve or improve β-cell function. These findings provide evidence and potential mechanisms for the role of obesity in promoting β-cell dysfunction, highlighting the potential importance of mitigating obesity and its metabolic effects in preventing and treating type 2 diabetes.

## Introduction

Type 2 diabetes mellitus (T2D) develops from progressive loss of pancreatic β-cell function on a background of chronic insulin resistance. The loss of β-cell compensation for insulin resistance occurs for years prior to the development of T2D [1]. Obesity is an important cause of insulin resistance and T2D [2]. Adipose tissue synthesizes and secretes a variety of hormones, collectively referred to as adipokines [3]. Circulating adipokines regulate biological processes in various target organs and tissues including pancreatic β-cells [4]. Thus, obesity and adipokines may directly and indirectly contribute to the progressive loss of β-cell function, leading to the development of T2D.

To date, only three limited longitudinal studies have examined the role of adiposity and adipokines on declining β-cell function over time [5–7]. They are all limited to no more than five adipokines. Two of these studies used surrogate measures of β-cell function derived from oral glucose tolerance testing [6, 7]. One of these three studies is our own study with limited sample size (n = 60) although β-cell function was derived from frequently sampled intravenous glucose tolerance testing (FSIGT) [5]. The objective of the present study is to assess the role of baseline and changes in three adiposity measures (BMI, percent body fat and trunk fat), insulin resistance and fifteen adipokines in relationship to change in β-cell function over time derived from repeated FSIGTs in a large prospectively collected longitudinal cohort of Mexican Americans (MA, n = 361) at risk for T2D.

## Materials and methods

### Data source

Data were from the BetaGene study, a family-based study of obesity, insulin resistance, and β-cell dysfunction in MA. Details regarding recruitment for the baseline [8] and follow-up [9] components of BetaGene have been described previously. Participants were recruited from Los Angeles County/University of Southern California Medical Center or Kaiser Permanente Southern California. Protocols for BetaGene were approved by the Institutional Review Boards of each institution, and all participants provided written informed consent prior to study enrollment.

A total of 370 subjects participated in baseline testing and repeat testing at a median (IQR) of 4.2 (3.4–5.5) years later. At each time, participants had a fasting blood draw to measure adipokines and a 75gm oral glucose tolerance test (OGTT). Participants with fasting glucose less than 126 mg/dL (7 mmol/l) were invited for a dual-energy x-ray absorptiometry (DXA) scan for body composition and insulin-modified FSIGT for measurement of insulin sensitivity and β-cell function. Briefly, dextrose (300 mg/kg body weight) was injected into an antecubital vein between 07:00–09:00 hrs. Insulin (0.03 U/kg body weight) was infused over five minutes starting 20 minutes after the glucose injection. A total of 21 arterialized venous blood samples were obtained from a heated hand vein between –15 and +240 minutes relative to the glucose injection. Plasma was separated immediately, stored at –80°C, and assayed for glucose and insulin. We report results from 361 participants for whom all 15 adipokines were measured successfully at baseline and follow-up.

### Adipokines

Fifteen circulating biomarkers were measured: adiponectin, IL-1β, IL-6, IL-1Ra, leptin, lipocalin, MCP-1, resistin, TNF-α, apelin, CRP, dipeptidyl peptidase-4 (DPP-IV), visfatin, secreted frizzled protein 4 (SFRP4) and secreted frizzled protein 5 (SFRP5). Adiponectin, IL-1β, IL-6, leptin, lipocalin, MCP-1, resistin and TNF-α were assayed using two Millipore multiplex kits with magnetic bead panels (Millipore, Billerica, MA) with assay sensitivity of 11, 0.5, 0.4, 4.7, 1.7, 1.1, 2.2 and 0.1pg/mL, respectively. ELISA was used to measure CRP (Millipore, Billerica, MA), apelin, DPP-IV, visfatin (Ray Biotech, Norcross, GA), IL-1Ra (AssayBiotech, Sunnyvale, CA), SFRP4 and SFRP5 (USCN Life Science, Wuhan, China) with assay sensitivity of 0.004 ng/mL, 29.1 pg/mL, 0.5 pg/mL, 0.78 ng/mL, 23 pg/mL, 26.6 pg/mL and 0.60 ng/mL, respectively. Intra- and inter-assay coefficients of variation for all adipokines are shown in S1 Table.

### Data analysis

Insulin sensitivity (SI) was derived from the minimal model analysis of FSIGTs[10]. Acute insulin response to glucose (AIRg) was calculated as the incremental area under the curve for insulin during the first 10 mins of the FSIGTs. β-cell function was estimated as the disposition index (DI = SI x AIRg), a measurement of β-cell compensation for insulin resistance.

Significance of changes in cohort characteristics were tested by Wilcoxon signed rank test. For regression analyses, log-transformation was applied to adiposity, adipokines, SI and DI, and rates of changes for these measures were derived as ([log of follow-up value—log of baseline value]/total follow-up time). Data were approximately normally distributed after the log-transformation. To make the regression coefficients directly comparable across different measures, we standardized each measurement by dividing each individual value by the cohort standard deviation (SD) prior to regression modeling. Thus, the regression coefficients are scale independent and represent change per SD in the dependent variable associated with change per SD in the independent variable. For bivariate analyses, both the baseline and rate of change for the same measurement were included in one model to assess independent contributions of baseline and change on the outcome. Multivariate analysis was then used to identify factors across adiposity, insulin resistance and/or the fifteen adipokines that were independently and significantly associated with rate of change in DI. Both forward and backward selection procedures were used in the multivariate analysis. Both selection procedures yielded same variables in the final model. Linear mixed-effect kinship regression models (LMKM) were used to account for relatedness adjusting for age, sex and kinship. All analyses were performed using R v.3.3.0[11]. All statistical tests were two-sided and statistical significance was defined as p<0.05.

## Results

Mean (±SD) age at baseline was 35±8 years; 73% of participants were female. Baseline and follow-up characteristics are provided in Table 1. At follow-up, BMI, percent body fat and trunk fat all increased significantly compared to baseline, as did fasting levels of CRP, IL-6, MCP-1, SFRP4, SFRP5. Circulating apelin, DPP-IV, lipocalin, and resistin decreased significantly. Adiponectin, IL-1β, IL-1Rα, leptin, TNF-α and visfatin did not change significantly over time. For glucose homeostasis, fasting and 2-hr glucose increased significantly during follow-up. The fraction of people with impaired glucose tolerance and diabetes increased from 33.0% and 3.9% to 35.7% and 9.4%, respectively, SI decreased significantly and AIRg did not change significantly, resulting in a significant decrease in DI. The associations of BMI, percent body fat and trunk fat with the fifteen adipokines at baseline and during follow-up are displayed in Table 2. Measures of adiposity at baseline or changes during follow-up were most strongly associated with levels of leptin, CRP, IL-1Ra and SFRP4 (all positive), and adiponectin and SFRP5 (all negative).

**Table 1:** Baseline and follow-up characteristics (n = 361)^a^.

	Baseline	Change during a median of 4.2 years of follow-up
Age (years)	34.6 (29.3, 40.3)	
Female (%)	265 (73.4%)	
*Anthropometrics*		
BMI [kg/m^2^]	28.6 (25.2, 32.6)	0.6 (-0.4, 1.7)^b^
Percent body fat [%]	36.1 (28.3, 40.2)	0.6 (-0.8, 2.1)^b^
Body trunk fat [kg]	12.7 (9.7, 16.8)	0.9 (-0.4, 2.0)^b^
*Adipokines*		
Adiponectin [μg/mL]	9.2 (6.3, 14.5)	-0.1 (-2.0, 1.8)
Apelin [ng/mL]	0.9 (0.5, 1.4)	-0.2 (-0.6, 0.2)^b^
CRP [ng/mL]	1.3 (0.6, 3.3)	0.1 (-0.4, 0.9)^b^
DPP-IV [ng/mL]	295 (224, 375)	-17 (-64, 23)^b^
IL-1β [pg/mL]	0.7 (0.6, 1.0)	0.0 (-0.1, 0.1)
IL-1Ra [pg/mL]	12.7 (7.6, 18.5)	-0.8 (-4.6, 3.1)
IL-6 [pg/mL]	3.0 (1.7, 5.3)	0.4 (-0.7, 1.8)^b^
Leptin [ng/mL]	13.6 (6.6, 25.6)	0.4 (-2.8, 4.0)
Lipocalin [ng/mL]	62 (51, 77)	-11 (-24, 1)^b^
MCP-1 [pg/mL]	111 (90, 139)	12 (-9, 35)^b^
Resistin [ng/mL]	19.6 (15.0, 27.4)	-0.8 (-3.6, 1.9)^b^
SFRP4 [ng/mL]	91.8 (63.2, 126.4)	9.9 (-3.6, 25.7)^b^
SFRP5 [ng/mL]	14.3 (8.5, 20.8)	1.8 (-1.0, 5.4)^b^
TNF-α [pg/mL]	3.3 (2.5, 4.5)	0.0 (-0.7, 0.6)
Visfatin [ng/mL]	15.6 (11.9, 22.0)	0.1 (-4.1, 6.9)
*Glucose homeostasis*		
Impaired glucose [%]*	119 (33.0%)	129 (35.7%)
Diabetes (%)*	14 (3.9%)	34 (9.4%)
Fasting glucose [mmol/L]	5.0 (4.7, 5.4)	0.1 (-0.3, 0.5)^b^
2-h glucose [mmol/L]	6.9 (5.8, 8.4)	0.4 (-0.6, 1.8)^b^
Insulin sensitivity [SI, x10^-3^ min^-1^ per pmol/L]	4.7 (3.2, 6.5)	-0.6 (-1.8, 0.4)^b^
Acute insulin response [AIRg, pmol/L x 10min]	2,828 (1,600, 4,544)	3 (-763, 799)
Disposition index [DI = SI x AIRg]	12,098 (7,992, 18,964)	-1,783 (-5,198, 1,344)^b^

*Diabetes is defined by fasting glucose ≥7.0 mmol/L or 2-hr glucose ≥11.11 mmol/L; Impaired glucose is defined as fasting glucose <7.0 mmol/L and 2-hr glucose ≥7.8 mmol/L but <11.11mmol/L

^a^ values shown are median (25^th^, 75^th^ percentile), unless otherwise noted

^b^ Significant at p<0.05 by Wilcoxon signed rank Test

**Table 2:** Standardized regression coefficients (p-values) between adiposity and adipokines at baseline and during follow-up (N = 361).

	Adiponectin	Apelin	CRP	DPP-IV	IL-1β	IL-1Ra	IL-6	Leptin	Lipocalin	MCP-1	Resistin	SFRP4	SFRP5	TNF-α	Visfatin
BMI															
Baseline^a^	**-0.363 (<0.001)**	**0.148 (0.005)**	**0.458 (<0.001)**	0.047 (0.37)	0.001 (0.98)	**0.328 (<0.001)**	**0.278 (<0.001)**	**0.698 (<0.001)**	**0.137 (0.009)**	0.101 (0.054)	0.073 (0.17)	**0.322 (<0.001)**	**-0.386 (<0.001)**	**0.133 (0.012)**	0.019 (0.72)
Follow-up^b^	**-0.354 (<0.001)**	0.049 (0.35)	**0.293 (<0.001)**	0.044 (0.41)	0.034 (0.52)	**0.199 (<0.001)**	**0.145 (0.006)**	**0.609 (<0.001)**	0.014 (0.79)	0.058 (0.27)	0.038 (0.47)	**0.161 (0.002)**	**-0.374 (<0.001)**	0.080 (0.12)	0.006 (0.91)
Follow-up^c^	**-0.336 (<0.001)**	0.040 (0.45)	**0.293 (<0.001)**	0.028 (0.59)	0.012 (0.81)	**0.162 (0.002)**	**0.129 (0.014)**	**0.591 (<0.001)**	0.032 (0.54)	0.046 (0.39)	0.046 (0.38)	**0.140 (0.008)**	**-0.344 (<0.001)**	0.055 (0.29)	-0.008 (0.87)
Percent body fat															
Baseline^a^	**-0.166 (0.002)**	**0.132 (0.012)**	**0.416 (<0.001)**	0.025 (0.63)	0.100 (0.058)	**0.338 (<0.001)**	**0.252 (<0.001)**	**0.673 (<0.001)**	**0.168 (0.001)**	0.082 (0.12)	**0.127 (0.016)**	**0.310 (<0.001)**	**-0.219 (<0.001)**	**0.166 (0.002)**	-0.046 (0.38)
Follow-up^b^	**-0.253 (<0.001)**	**0.133 (0.011)**	**0.253 (<0.001)**	0.000 (0.99)	0.021 (0.68)	**0.317 (<0.001)**	**0.125 (0.018)**	**0.534 (<0.001)**	**0.113 (0.033)**	0.099 (0.06)	0.100 (0.058)	**0.155 (0.003)**	**-0.299 (<0.001)**	0.050 (0.35)	-0.090 (0.088)
Follow-up^c^	**-0.246 (<0.001)**	**0.116 (0.028)**	**0.253 (<0.001)**	-0.028 (0.60)	-0.028 (0.59)	**0.282 (<0.001)**	0.089 (0.092)	**0.511 (<0.001)**	**0.126 (0.017)**	0.089 (0.09)	0.100 (0.056)	**0.116 (0.028)**	**-0.268 (<0.001)**	0.009 (0.87)	**-0.121 (0.021)**
Trunk Fat															
Baseline^a^	**-0.371 (<0.001)**	**0.143 (0.007)**	**0.449 (<0.001)**	0.034 (0.53)	0.047 (0.38)	**0.387 (<0.001)**	**0.253 (<0.001)**	**0.714 (<0.001)**	**0.143 (0.006)**	0.078 (0.14)	**0.120 (0.022)**	**0.324 (<0.001)**	**-0.408 (<0.001)**	**0.161 (0.002)**	0.000 (0.99)
Follow-up^b^	**-0.294 (<0.001)**	**0.155 (0.003)**	**0.283 (<0.001)**	0.021 (0.69)	0.050 (0.34)	**0.304 (<0.001)**	**0.137 (0.009)**	**0.600 (<0.001)**	0.088 (0.095)	0.100 (0.058)	0.068 (0.19)	**0.167 (0.001)**	**-0.370 (<0.001)**	0.053 (0.32)	-0.064 (0.22)
Follow-up^c^	**-0.282 (<0.001)**	**0.134 (0.011)**	**0.292 (<0.001)**	-0.020 (0.70)	0.009 (0.87)	**0.263 (<0.001)**	**0.117 (0.027)**	**0.580 (<0.001)**	**0.113 (0.031)**	0.088 (0.097)	0.081 (0.13)	**0.140 (0.008)**	**-0.332 (<0.001)**	0.017 (0.74)	-0.099 (0.061)

Significant correlations are denoted in bold font

^a^ Adjusted for age and sex

^b^ Rates of change during follow-up were used in calculating the correlation coefficients, adjusted for age and sex at baseline

^c^ Rates of change during follow-up were used in calculating the correlation coefficients, adjusted for age, sex and the adiposity trait at baseline

In the bivariate analyses (Table 3), rate of change in DI was negatively associated with all three baseline measures of adiposity, although the association was not statistically significant for baseline percent body fat. Among the fifteen adipokines, both baseline level and rate of change in adiponectin, and baseline level and rate of change in SFRP5 were positively associated with rate of change in DI. Rates of change in CRP, IL-6, and leptin were negatively associated with rate of change in DI. Increasing BMI, followed by increasing CRP and trunk fat, were most strongly associated with decreasing DI over time. Rates of change in all three adiposity measures, as well adiponectin, CRP, IL-1Ra, IL-6, leptin, and SFRP5 were all negatively associated with rate of change in SI (Table 3). Increasing BMI, percent body fat and trunk fat, followed closely by increasing leptin, were most strongly associated with decreasing SI.

**Table 3:** Bivariate associations between rate of change in disposition index (DI) and insulin sensitivity (SI) with each of the adiposity or adipokine measures*.

Variables	Rate of Change in DI		Rate of Change in SI	
	Beta	P-value	Beta	P-value
*Adiposity*				
BMI				
Baseline	**-0.109**	**0.041**	0.050	0.33
Rate of change	**-0.250**	**<0.001**	**-0.415**	**<0.001**
Percent body fat				
Baseline	-0.098	0.24	0.028	0.73
Rate of change	**-0.146**	**0.010**	**-0.349**	**<0.001**
Body trunk fat				
Baseline	**-0.139**	**0.021**	0.007	0.90
Rate of change	**-0.196**	**0.001**	**-0.366**	**<0.001**
*Adipokines*				
Adiponectin				
Baseline	**0.156**	**0.005**	0.070	0.22
Rate of change	**0.121**	**0.025**	**0.166**	**0.002**
Apelin				
Baseline	-0.067	0.25	0.001	0.98
Rate of change	0.029	0.63	0.023	0.70
C-reactive protein				
Baseline	-0.078	0.21	0.071	0.25
Rate of change	**-0.199**	**0.001**	**-0.140**	**0.016**
DPP-IV				
Baseline	0.108	0.071	0.109	0.065
Rate of change	-0.002	0.97	0.038	0.53
IL-1β				
Baseline	0.087	0.11	-0.010	0.86
Rate of change	0.023	0.66	-0.081	0.13
IL-1Ra				
Baseline	-0.012	0.83	0.038	0.50
Rate of change	-0.051	0.37	**-0.157**	**0.004**
IL-6				
Baseline	0.000	0.99	0.038	0.50
Rate of change	**-0.154**	**0.007**	**-0.173**	**0.002**
Leptin				
Baseline	-0.055	0.42	0.049	0.45
Rate of change	**-0.175**	**0.002**	**-0.343**	**<0.001**
Lipocalin				
Baseline	0.036	0.53	0.055	0.34
Rate of change	0.017	0.76	0.011	0.85
MCP1				
Baseline	-0.009	0.88	0.053	0.35
Rate of change	-0.044	0.44	0.029	0.61
Resistin				
Baseline	0.055	0.31	0.051	0.35
Rate of change	-0.006	0.92	-0.002	0.98
SFRP4				
Baseline	-0.074	0.21	-0.013	0.83
Rate of change	-0.039	0.50	-0.054	0.34
SFRP5				
Baseline	**0.132**	**0.017**	0.051	0.37
Rate of change	**0.128**	**0.016**	**0.249**	**<0.001**
TNFα				
Baseline	0.033	0.56	0.025	0.66
Rate of change	-0.079	0.16	-0.050	0.37
Visfatin				
Baseline	0.067	0.29	0.029	0.65
Rate of change	0.049	0.44	0.057	0.37

*Beta represents change per standard deviation (SD) in dependent variables (row) associated with change per SD in independent variables (column) adjusting for age, sex and kinship. For the same independent variables in the first column, baseline and rate of change were included simultaneously in one model to assess the independent associations with baseline and change

Significant associations were denoted in bold font.

In the multivariate analyses, three variables were independently and significantly associated with rate of change in DI (Table 4). The strongest was rate of change in BMI (negative, p<0.001), followed by baseline adiponectin (positive, p = 0.004), and rate of change in IL-6 (negative, p = 0.011). Note that rates of change in CRP, leptin and SFRP5 were not significantly associated with rate of change in DI in the multivariate analysis. These measures were highly correlated with change in BMI (see Table 2), the inclusion of which eliminated them from significance in the multivariate model. Baseline SFRP5 was no longer significant due to its high correlation with baseline adiponectin (r = 0.78).

**Table 4:** Significant multivariate associations with rate of change in DI^a^.

Variables in the final model	Adjusted for rate of change in SI			
	No		Yes	
	Beta ± SE	P-value	Beta ± SE	P-value
Rate of change in BMI	-0.216 ± 0.051	<0.001	0.009 ± 0.049	0.86
Baseline adiponectin	0.150 ± 0.052	0.004	0.111 ± 0.045	0.015
Rate of change in IL-6	-0.130 ± 0.051	0.011	-0.052 ± 0.045	0.25

^a^ Beta presents change per standard deviation (SD) in DI associated with change per SD for the variables in the first column after adjusting for baseline age, sex, kinship and other variables in the first column without or with further adjusting for rate of change in SI

Since we have previously shown that increasing insulin resistance is an important component contributing to declining β-cell function, we examined whether the observed associations with longitudinal change in DI were due to change in insulin resistance by further including rate of change in SI as a covariate in the model (see right two columns in Table 4). This adjustment eliminated the association between rate of change of DI and rates of change in BMI and IL-6. By contrast, the association between rate of change in DI and baseline adiponectin levels remained significant after adjustment for rate of change in SI.

## Discussion

In this Mexican-American cohort observed for over 4 years, weight and adiposity significantly increased over time. Such increases were accompanied by a variety changes in adipokines (increasing CRP, IL-6, MCP-1, SFRP4, SFRP5, and decreasing apelin, DPP-IV, lipocalin and resistin) and glucose homeostasis measures (increasing fasting and 2-hr glucose, and decreasing insulin sensitivity and β-cell function). Examining the role of adiposity and adipokines in β-cell function decline over time, we found that three factors were significantly and independently associated with rate of change in DI over time. They were rate of change in BMI (negative), baseline adiponectin (positive) and rate of change in IL-6 (negative). The association with change in DI was the strongest for changing BMI and appeared to be explained in large part by changing insulin resistance, in keeping with prior observations on the contributions of insulin resistance to declining beta cell function [5] and treatment of insulin resistance to preserve β-cell function and prevent type 2 diabetes [12, 13]. An association between changing IL-6 and changing DI was also largely mitigated by adjustment for changing insulin resistance. Baseline adiponectin remained positively associated with changing DI after adjustment for changing SI, suggesting an independent effect of adiponectin to preserve or improve beta cell function.

The most novel finding in this study was that high levels of baseline adiponectin were associated with less reduction in β-cell function over time in this observational Mexican-American cohort at risk for type 2 diabetes, independent of weight gain, insulin resistance and fourteen other adipokines. Adiponectin is one of the commonly studied adipokines and many groups have demonstrated association with obesity and type 2 diabetes. The Diabetes Prevention Program trial showed that high baseline adiponectin levels in people with pre-diabetes were associated with lower risk of type 2 diabetes independent of weight gain [14]. Our findings advanced knowledge compared to prior studies by demonstrating a protective effect of adiponectin on β-cell compensation during follow-up independent of insulin resistance and weight gain.

The potential roles of the protective effect of adiponectin on beta cell function may be through at least two mechanisms. First, it is known that adiponectin increases insulin sensitivity, as shown in this study (Table 3) as well as others [15, 16]. This increase in sensitivity may unload the beta cells and slow the loss of beta-cell function over time. Indeed, we observed that adjustment for change in insulin sensitivity partly, but not completely explained the association between baseline adiponectin and change in DI (Table 4). Second, adiponectin may have direct effects on beta cells through reducing apoptosis and promoting beta cell expansion, as shown in pre-clinical studies [17] [18].

Of the fifteen adipokines we studied, fourteen were not significantly and independently associated with beta cell function decline after adjusting for weight gain and change in insulin sensitivity. Of the fourteen adipokines, changes in four adipokines (CRP, IL-6, leptin and SFRP5) were associated with change in DI in the bivariate analysis. The four were also associated with changes in insulin sensitivity and weight gain. SFRP5 was highly correlated with adiponectin [19]; both forward and backward multivariate analysis showed that adiponectin has a stronger association with change in DI than SFRP5 such that its association with DI was eliminated after adiponectin was included in the model. The role of SFRP5 on insulin sensitivity and beta cell function has been controversial [20] [21]. It is well known that CRP and leptin are strongly associated with increasing adiposity [19]. Inclusion of weight gain eliminated their significant associations with change in DI, suggesting that they may mediate some of the detrimental effects of obesity on beta cell function, as we observed in Hispanic women with a history of gestational diabetes [5]. The non-significant association between IL-6 and DI after adjustment for change in insulin sensitivity is consistent with prior reports that IL-6 contributes to the development of insulin resistance [22]. We did not find associations between the remaining ten adipokines (apelin, DPP-IV, IL-1β, IL-1Rα, lipocalin, MCP1, resistin, SFRP4, TNFα and visfatin) and change in DI. Of them, IL-1Rα has been tested and found not to be associated with change in beta cell function measured by oral glucose tolerance testing, although it was associated with change in insulin sensitivity [7], as we found (Table 3). Thus, our results on CRP, leptin, IL-6 and IL-1Rα were consistent with previous reports. To our knowledge, no previous studies have assessed associations of apelin, DPP-IV, IL-1β, ipocalin, MCP1, resistin, SFRP4, TNFα and visfatin on change in beta cell function over time in humans.

Overall, our results provide evidence and potential mechanisms for the role of obesity in promoting β-cell dysfunction and diabetes, as well as highlighting the potential importance of mitigating obesity and its metabolic effects in preventing and treating type 2 diabetes in Mexican Americans.

## Supporting information

S1 Table Intra- and inter- assay coefficients of variation (CV). (DOCX)

## Abbreviations

AIRg: Acute insulin response to glucose
DPP-IV: Dipeptidyl peptidase-4
DI: Disposition index
DXA: Dual-energy x-ray absorptiometry
FSIGT: Frequently sampled intravenous glucose tolerance testing
SI: Insulin sensitivity
IQR: Interquartile range
MA: Mexican Americans
OGTT: Oral glucose tolerance test
SFRP4: Secreted frizzled protein 4
SFRP5: Secreted frizzled protein 5
SD: Standard deviation
T2D: Type 2 diabetes mellitus
