# Supplementary material for: Association of weight gain and fifteen adipokines with declining beta-cell function in Mexican Americans
Source: PLoS One. 2018 Aug 13;13(8):e0201568. doi: 10.1371/journal.pone.0201568 (PMC6089433; doi:10.1371/journal.pone.0201568)
Supplement: S1 Table — (DOCX) [file pone.0201568.s001.docx]

S1 Table. Intra- and inter-assay coefficients of variation (CV)

| **Analytes** | **Intra-assay CV** | **Inter-assay CV** |
| --- | --- | --- |
| **Adipokine Panel 1 (3-plex):** |  |  |
| Adiponectin | 1.66% | 11.3% |
| Lipocalin | 3.3% | 13.7% |
| Resistin | 3.06% | 21.8% |
| **Adipokine Panel 2 (5 plex):** |  |  |
| IL-1β | 5.25% | 18.4% |
| IL-6 | 4.75% | 9.2% |
| MCP-1 | 8.73% | 10.3% |
| Leptin | 5.16% | 7.0% |
| TNF-α | 6.83% | 11.0% |
| CRP | 7.09% | 5.4% |
| IL-1Ra | 8.82% | 10.3% |
| DPP-IV | 9.17% | 7.9% |
| Apelin | 16.77% | 5.3% |
| SFRP4 | 6.69% | 21.6% |
| SFRP5 | 7.46% | 7.0% |
| Visfatin | 5.88% | 5.1% |
